# Ultrafast energy-dispersive soft-x-ray diffraction in the water window with a laser-driven source

**DOI:** 10.1063/4.0000270

**Published:** 2024-10-11

**Authors:** Jasmin Jarecki, Martin Hennecke, Themistoklis Sidiropoulos, Matthias Schnuerer, Stefan Eisebitt, Daniel Schick

**Affiliations:** 1 Max-Born-Institut für Nichtlineare Optik und Kurzzeitspektroskopie, Max-Born-Straße 2A, 12489 Berlin, Germany; 2 Technische Universität Berlin, Institut für Optik und Atomare Physik, 10623 Berlin, Germany

## Abstract

Time-resolved soft-x-ray-diffraction experiments give access to microscopic processes in a broad range of solid-state materials by probing ultrafast dynamics of ordering phenomena. While laboratory-based high-harmonic generation (HHG) light sources provide the required photon energies, their limited photon flux is distributed over a wide spectral range, rendering typical monochromatic diffraction schemes challenging. Here, we present a scheme for energy-dispersive soft-x-ray diffraction with femtosecond temporal resolution and photon energies across the water window from 200 to 600 eV. The experiment utilizes the broadband nature of the HHG emission to efficiently probe large slices in reciprocal space. As a proof-of-concept, we study the laser-induced structural dynamics of a Mo/Si superlattice in an ultrafast, non-resonant soft-x-ray diffraction experiment. We extract the underlying strain dynamics from the measured shift of its first order superlattice Bragg peak in reciprocal space at photon energies around 500 eV via soft-x-ray scattering simulations.

## I. INTRODUCTION

Soft-x-ray radiation is highly sensitive to a wide range of phenomena in physics, chemistry, biology, and materials science. By covering core-to-valence resonances, it can spectroscopically probe charge, spin, and orbital degrees of freedom in various material systems with element sensitivity.^1,2^ For solid-state research, the short wavelengths also enable few-nanometer spatial resolution, e.g., via diffraction and imaging techniques,^3–7^ and exhibit considerable penetration depth, rendering soft-x-ray techniques eligible for studying nanoscale heterostructures and buried layers.^8–10^ In addition to accelerator-based installations at free-electron lasers and synchrotron-radiation facilities, laser-driven high-harmonic generation (HHG) is becoming increasingly popular as a source of short-wavelength radiation spanning from the extreme ultraviolet (XUV)^11–13^ into the water-window, ranging from the C K-edge at 284 eV up to the O K-edge at 531 eV and beyond.^14,15^ The HHG's properties, such as spectrum, polarization, and pulse duration, are directly controlled by the driving laser and medium,^16,17^ facilitating the application of diverse experimental techniques with a temporal resolution down to the attosecond regime.^18–21^ The key to reaching the soft-x-ray photon energy range by HHG is to increase the driving laser wavelength $\lambda_{l}$, which at the same time leads to a severe decrease in the HHG efficiency scaling with $\lambda_{l}^{-(5-6)}$.^22–24^ Furthermore, the achievable, comparatively small photon flux is also distributed over a broad spectral range, typically spanning hundreds of electron volts (eV).^25–27^ This broadband emission renders HHG sources in the soft-x-ray range ideally suited for broadband absorption spectroscopy techniques, which have been successfully demonstrated in a series of ground-breaking experiments mainly targeting local atomic and molecular dynamics.^28–31^ Diffraction experiments, however, which can provide access to nanoscale long-range order, typically require extremely photon-inefficient monochromatization of the broad HHG spectra.^32^ As a result, time-resolved diffraction experiments within the water window employing HHG sources have been elusive so far.

Here, we show how to benefit from the broadband and quasi-continuous nature of HHG spectra in soft-x-ray diffraction experiments by realizing an energy-dispersive scheme^33,34^ for efficiently probing large slices in reciprocal space. We utilize femtosecond, broadband soft-x-ray pulses ranging from 200 to 600 eV to enable time-resolved diffraction experiments at around 500 eV for the first time at an HHG-based setup. For this proof-of-concept experiment, we study the photoinduced structural dynamics of a Mo/Si superlattice (SL) in non-resonant, specular diffraction geometry. We observe a shift of the SL's first order Bragg peak in reciprocal space on a few picosecond timescale. A combination of ultrafast thermo-elastic and x-ray-scattering simulations can fully reproduce these experimental results, enabling direct and quantitative access to the underlying coherent motion of the atomic lattice. Our findings demonstrate the feasibility and sensitivity of the energy-dispersive diffraction approach, especially for probing Bragg peak shifts in reciprocal space, independent of the HHG pulse-to-pulse intensity fluctuations. Extending the applicability of HHG-driven soft-x-ray sources in this high photon-energy range will enable a variety of time-resolved diffraction experiments on ordering phenomena of electronic, structural, and magnetic origin.^35–38^

## II. RESULTS AND DISCUSSION

**FIG. 1.**
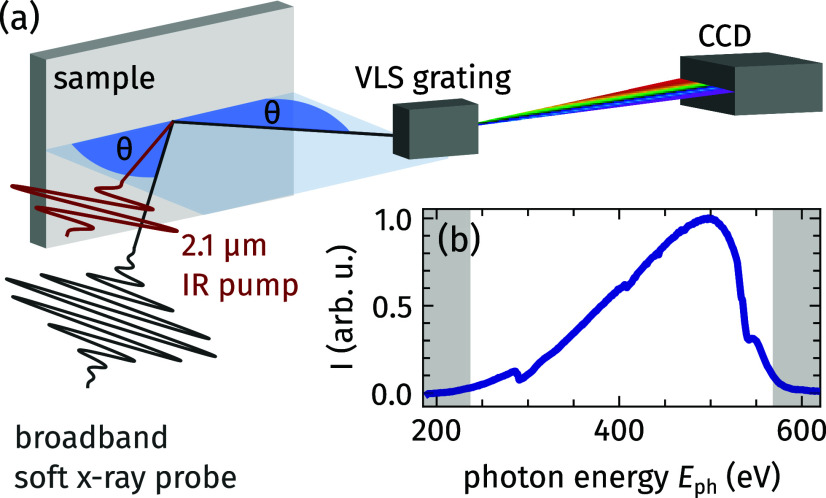
(a) Setup for time-resolved energy-dispersive soft-x-ray diffraction utilizing an HHG source. The broadband soft-x-ray pulses hit the sample at a variable grazing angle θ and are scattered specularly off the sample. The rotatable spectrometer combines a variable-line-spacing (VLS) grating and a CCD camera to detect scattered soft-x-ray radiation with photon energy resolution. The 2.1 μm-MIR pulses excite the sample quasi-collinearly with the probe pulses. (b) Typical soft-x-ray spectrum emitted by the HHG source ranging from 200 to 600 eV. The gray areas mark the spectral regions, which are omitted in the analysis due to insufficient photon flux.

The general concept of our soft-x-ray diffraction experiment, combining a common θ/2θ diffractometer with a spectrometer,^39^ is sketched in Fig. 1(a). As HHG driver, we employ a high-average-power mid-infrared (MIR) optical parametric chirped pulse amplifier (OPCPA) system operating at a repetition rate of 10 kHz providing pulses of 27 fs (full width at half maximum, FWHM) duration at a central wavelength of 2.1 μm.^40^ The laser pulses are focused into a He gas cell at a backing pressure of 2.3 bar and subsequently generate soft-x-ray pulses of ≤27 fs FWHM duration spanning a broad spectral range from 200 to 600 eV. The soft-x-ray probe pulses impinge on the sample under a grazing angle θ and get scattered into the rotatable spectrometer at an angle of 2θ with respect to the incident beam. The spectrometer consists of a variable-line-spacing (VLS) grating and an in-vacuum CCD camera, which provide a spectral resolution of about 1 eV at a photon energy, $E_{\text{ph}}$, of 500 eV. A typical soft-x-ray spectrum after transmission through two Al filters of a total thickness of 500 nm fully covering the water window is depicted in Fig. 1(b). Absolute photon numbers have been determined with calibrated detection equipment previously.^41^ In the spectrum, several absorption lines corresponding to K-edges of residual molecules of C (285 and 292 eV^42^), N (409.9 eV^15^), and O (531 and 538 eV^43^) can be identified and utilized for energy calibration of the spectrometer. A 8%-fraction of the 2.1 μm laser light is split off and guided via a mechanical delay line to the sample for photoexcitation. A rotatable waveplate and polarizer set the incident fluence to 9 mJ/cm^2^. Due to the non-collinearity of 3.3° between the pump and the probe pulses and their spot sizes at normal incidence of 543 × 317 μm^2^ FMWH and 77 × 111 μm^2^ FMWH, respectively, we achieve a temporal resolution of about 40 fs. Both the pump and probe pulses are *p*-polarized with respect to the scattering plane.

In a diffraction experiment probing the sample's out-of-plane (OOP) order along the *z*-direction, the magnitude of the corresponding scattering vector, $Q_{z}=|\vec{Q}|=|{\vec{k}}_{\text{out}}-{\vec{k}}_{\text{in}}|$, is defined as

$$Q_{z}=\frac{4\pi \;E_{\text{ph}}}{h\;c_{0}}\text{sin}\;\theta ,$$ (1)

where *h* denotes the Planck constant and $c_{0}$ is the speed of light in vacuum. In order to access an OOP periodicity *d*, a multiple of its corresponding reciprocal lattice vector $|\vec{G}|=\frac{2\pi }{d}$ must match the scattering vector $\vec{Q}$, as described by the Laue condition,

$$\vec{Q}={\vec{k}}_{\text{out}}-{\vec{k}}_{\text{in}}=n\vec{G}.$$ (2)

Here, ${\vec{k}}_{\text{in}/\text{out}}$ denotes the wavevectors of the incident and scattered soft-x-ray light, respectively, and *n* is an integer number corresponding to the diffraction order. Typical monochromatic diffraction schemes comprise time-consuming variations of θ and/or $E_{\text{ph}}$ for scanning reciprocal space, rendering themselves inconvenient and inefficient for broadband HHG sources in the water window. The concept of probing diffraction in an energy-dispersive mode circumvents this requirement and enables access to large reciprocal-space volumes very photon- and time-efficiently in a single acquisition (see the supplementary material).^63^

**FIG. 2.**
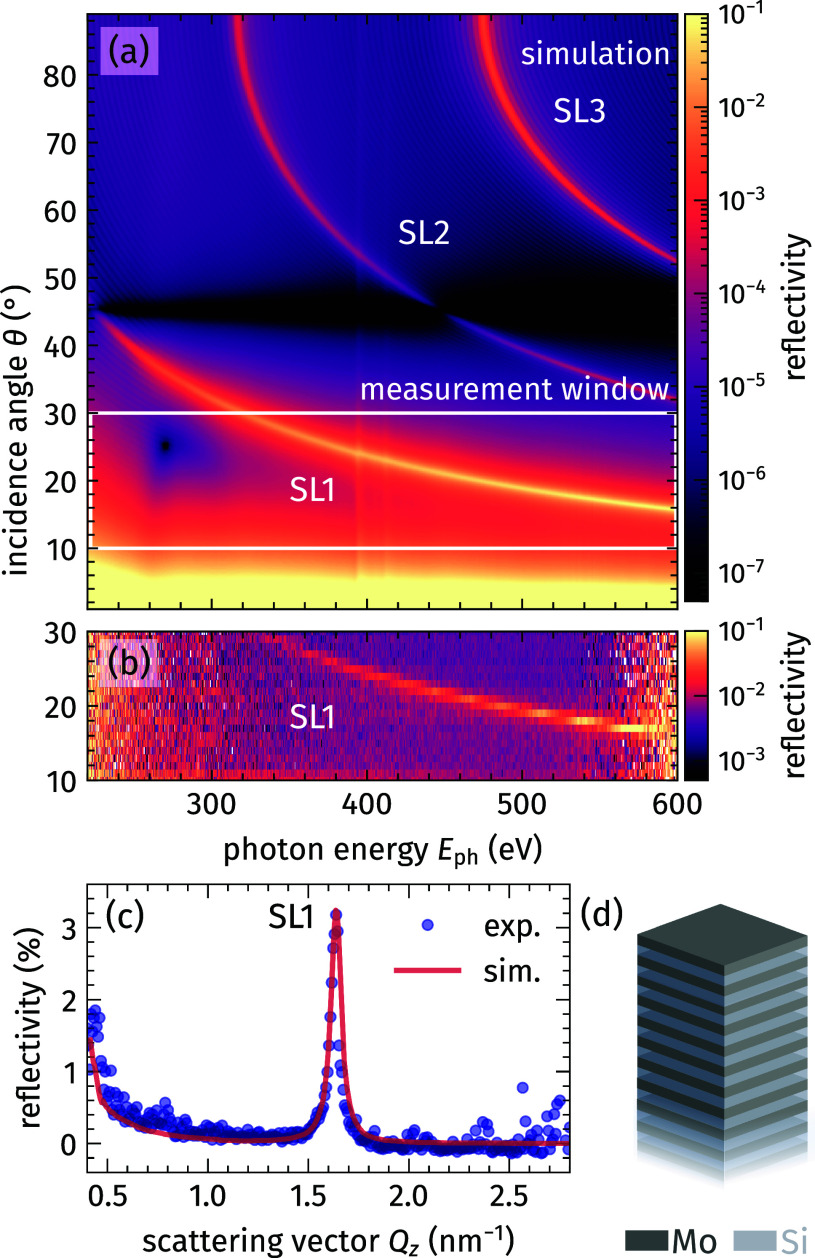
Energy-resolved soft-x-ray diffraction from the Mo/Si superlattice (SL) structure. (a) Simulated x-ray-scattering signal of the Mo/Si SL structure [see panel (d)] with varying incidence angle θ and photon energy $E_{\text{ph}}$. Soft-x-ray light diffracted from the periodically ordered Mo/Si layers results in distinct intensity peaks, i.e., SL Bragg peaks of order *n* (SL*n*). (b) Measurement of the diffraction signal employing broadband soft-x-ray pulses under variation of the incidence angle θ from 10° to 30° as highlighted by the white box in panel (a). (c) The simulated and experimental data from (a) and (b) are transformed into reciprocal space, revealing the first order SL Bragg peak.

To demonstrate the capabilities of the approach and experimental realization, we study the laser-induced structural dynamics of a Mo/Si superlattice, a material system that allows to realize efficient mirrors in the XUV and soft-x-ray spectral range.^44–46^ The particular sample structure presented in this paper has already been investigated in a previous study, focusing on its use as a cross-correlator for optical laser pulses with soft-x-ray pulses at a broad range of photon energies.^47^ The SL is built of 40 double layers (DL) of 1.88 nm polycrystalline Mo and 2.05 nm amorphous Si on a crystalline Si substrate [see Fig. 2(d)]. The artificial unit cell of the SL is defined by the double-layer thickness $d_{\text{DL}}=3.93\;\text{nm}$ and the corresponding reciprocal lattice vector $G_{\text{DL}}=2\pi /d_{\text{DL}}$. The structural parameters have been extracted and refined by comparing static synchrotron data with matrix-formalism-based x-ray-scattering simulations^48,49^ (see the supplementary material).^63^

We use the same simulations to model the x-ray-scattering signal from the Mo/Si SL in our energy-dispersive setup in Fig. 2(a). We show the static result for the same angular and photon-energy range accessible in the experiment, which in principle gives access to three SL Bragg peaks of the order n=1,2,3. While the SL Bragg peaks appear at fixed scattering vectors $\vec{Q}$ in reciprocal space, they can be accessed by different combinations of the incidence angle θ and the photon energy $E_{\text{ph}}$ in real-space coordinates according to Eq. (1). In Fig. 2(b), we show the experimental, energy-dispersed scattering intensity for incidence angles θ in the range of 10°–30° with a step size of 1°. The experimental spectra are obtained from 5 s-integrations for each angle, resulting in a reasonably short acquisition time of the total scan. Based on the high spectral stability of our HHG source, we can normalize the experimental data of a total angle scan to the source spectrum as shown in Fig. 1(b). As a result, the experimental data are intrinsically self-normalized within every acquisition, providing accurate *relative* intensities of diffraction signals. However, because we lack an online monitor for the integrated intensity of the HHG, we do not rely on the *absolute* amplitude of the diffraction signal. Instead, we scale all acquired spectra of a scan to match the absolute intensities of the reliable x-ray-scattering simulations of the high-quality Mo/Si SL. This procedure does not influence the SL Bragg positions. The measured data agrees very well with the expected shift of the SL1 Bragg peak in photon energy with varying θ. This static result already highlights the sensitivity and efficiency of the energy-dispersive diffraction approach to capture large slices in reciprocal space in a single acquisition.

For a quantitative comparison of the simulation and experimental data from panels (a) and (b) of Fig. 2, we convert both datasets into reciprocal space, following Eq. (1). This transformation for all photon energies at every θ position results in a diffraction profile with a Bragg peak appearing at the same specific scattering vector $Q_{z}$. To enable proper averaging of the diffraction data along the θ-axis, it is necessary to map the data of each scan onto the same regular $Q_{z}$-grid, as scanning the incidence angle leads to a shift and a different spacing of the probed reciprocal-space volume.^50^ In Fig. 2(c), we show the averaged experimental data (blue symbols) together with the simulation (red solid line) after the $Q_{z}$-transformation. In the analysis of both the experimental data and the simulation, we consider only the spectral range of sufficiently high soft-x-ray intensity in the experiment between 230 and 570 eV [non-shaded area in Fig. 1(b)]. We achieve an excellent agreement between experiment and simulation by adjusting only the absolute angle of incidence θ by a small offset of 0.5° and scaling the measured intensities by a single factor for all spectra as described above. We observe the first order SL Bragg peak at $Q_{z}=1.626\;{\text{nm}}^{-1}$, which is clearly shifted with respect to the predicted value following the Laue condition, c.f. Eq. (2), at $Q_{z}^{\text{Laue}}=G_{\text{DL}}=1.599\;{\text{nm}}^{-1}$. It has been shown that this offset can be attributed to refraction effects introducing different phase shifts between interfering wavefronts as compared to the hard-x-ray range, resulting in the *shifted* Bragg peak position.^8,51–53^

Next, we measure the laser-induced response of the Mo/Si SL in a pump-probe experiment with femtosecond temporal resolution. The laser-driven dynamics of the Mo/Si SL can be expected to evolve as follows:^47^ The laser energy is mainly absorbed by the metallic Mo layers, whereas the semiconductor Si is transparent for the 2.1 μm-pump-laser pulses. This results in an alternating pattern of quasi-instantaneously heated and cold SL layers with an exponentially decaying excitation amplitude along the depth of the sample. The corresponding penetration depth of the pump laser light is of about 25 nm (see the supplementary material).^63^ This ultrafast heating leads to a rapid expansion of the Mo sub-lattice, while the cold Si layers get compressed, leading to a complex pattern of zone-folded longitudinal acoustic phonons (ZFLAPs)^54,55^ within the SL. On top of this sub-ps oscillatory dynamics, a bipolar strain wave is launched from the surface of the SL toward its lower interface.^56–58^ The picosecond timescale of this coherent strain-wave propagation is unambiguously determined by the acoustic sound velocity of about 6.2 nm/ps in Mo and 8.2 nm/ps in Si and by the thickness of the Mo/Si SL. Simultaneously, Si also starts to expand once energy is transferred via phononic heat diffusion between the few-nanometer-thin layers of Mo and Si, resulting in an increased double-layer thickness according to the laser-induced temperature rise in both materials. On nanosecond timescales, the SL starts to relax back to its equilibrium via heat transport to the substrate.^47,59^

**FIG. 3.**
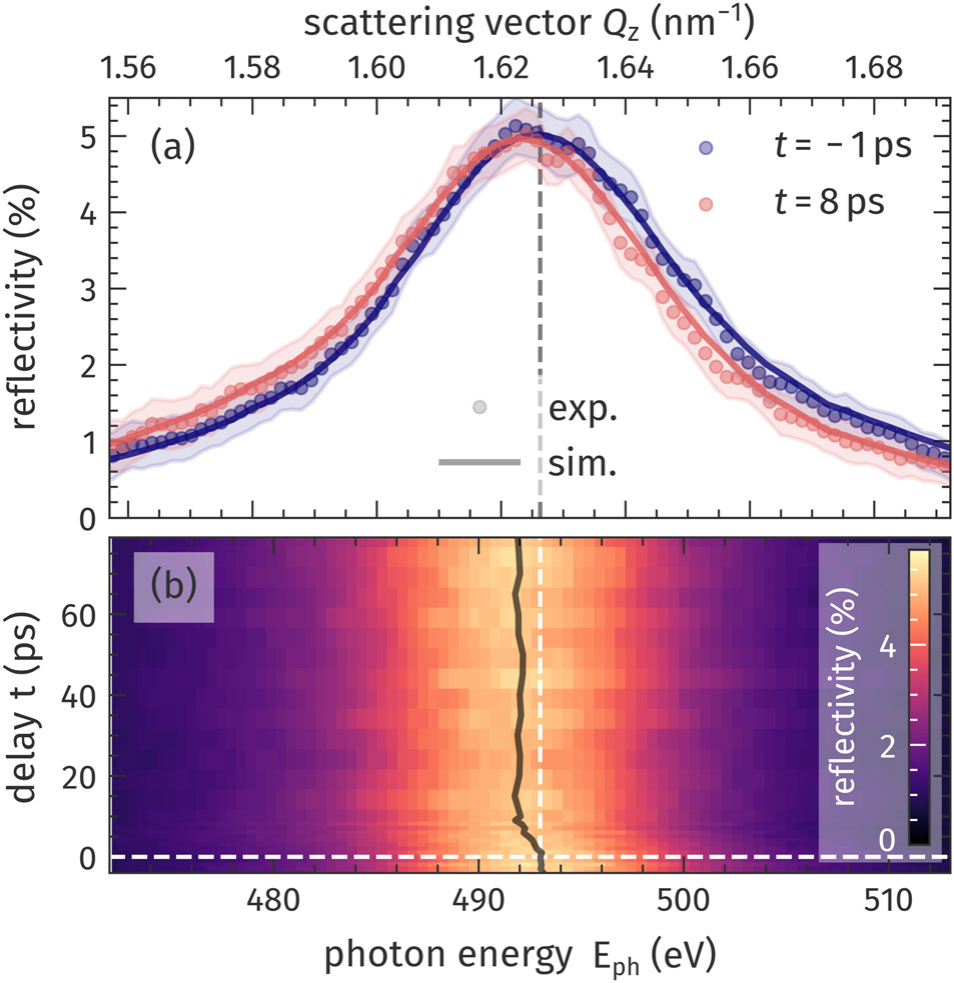
Transient energy-dispersive diffraction from the Mo/Si superlattice. (a) The first order SL Bragg peak before and after laser excitation shifts along $Q_{z}$ in reciprocal space or equivalently in energy in real space. Solid lines represent the modeled x-ray-scattering signal and circles the experimental data at a fixed angle of incidence θ=19°. The shaded area is defined by the standard error of each data point representing the uncertainty of the measurement at both delays. (b) Full map of energy-dispersive reciprocal-space scans for pump-probe delays ranging from −4 to 79 ps. The extracted Bragg peak position (black solid line) clearly reveals even small transient shifts in reciprocal space. Both panels share the scattering vector and photon-energy axis.

In the transient, energy-dispersive diffraction experiment, we probe the evolution of the SL1 Bragg peak at a fixed incidence angle of θ=19° to particularly follow the propagation of the coherent longitudinal strain wave described above. Sub-picosecond oscillations in the intensity of the first SL Bragg peak due to ZFLAPs are expected to be only of the order of 0.5%, which we are currently not able to resolve limited by the signal-to-noise ratio of the absolute intensity in our experiment. The presented delay scan comprises 12 loops of 30 s integration at each delay point resulting in a total measurement duration of about 4 h. The experimental data, as shown in Fig. 3(a), reveal an increasing transient shift of the SL1 Bragg peak to smaller scattering vectors $Q_{z}$ or smaller photon energies $E_{\text{ph}}$. This shift can be qualitatively understood as a direct translation of the laser-driven lattice expansion into reciprocal space via the Laue condition [Eq. (2)]. For the complete transient dataset, as shown in panel (b), we extract the average SL1 Bragg peak position by Gaussian fits (solid black line). To gain quantitative insights into the laser-driven lattice dynamics, we use the udkm1Dsim toolbox^49^ to simulate the thermo-elastic response of the Mo/Si SL. To that end, we first calculate the pump laser's absorption profile according to the multi-layer absorption formalism to determine the initial spatial energy distribution. In the second step, we use a Fourier heat-diffusion model to calculate the spatiotemporal temperature rise in the sample. This temperature map can be used to obtain the transient strain response by solving a linear-chain model of masses and springs. The details and parameters of these simulations are given in the supplementary material.^63^ In the final step, the structural dynamics are fed as input into the same x-ray-scattering formalism, which has been benchmarked by the static HHG and synchrotron data [Fig. 2(a)] to model the transient, energy-dispersive x-ray-diffraction signal. Using literature values for the thermo-elastic sample parameters (see supplementary material)^63^ and with the laser-excitation fluence as the only free parameter, we can fully simulate the shift of the SL1 Bragg peak, as shown by the solid lines in Fig. 3(a) for the two selected delays.

**FIG. 4.**
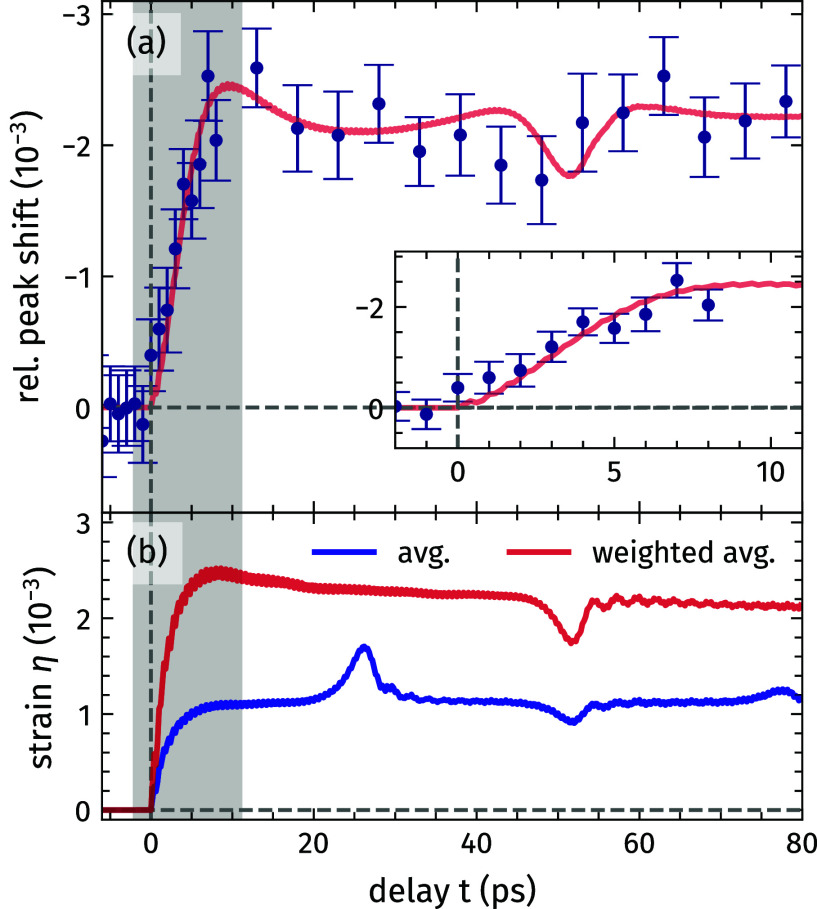
Temporal evolution of the lattice dynamics within the Mo/Si superlattice. (a) The extracted relative shift of the SL1 Bragg peak from the experimental (symbols) and simulated (solid line) x-ray diffraction. The inset displays a detailed view on the initial lattice dynamics marked by the shaded area. The error bars correspond to the statistical standard error of the mean value of the extracted peak position for each scan at each delay. (b) Simulated average strain of the full Mo/Si SL (blue line) and weighted by the soft-x-ray absorption profile at 500 eV (red line).

In Fig. 4(a), we show the full temporal evolution of the relative SL1 Bragg peak position ΔE/E(t<0 ps) extracted from both the transient experimental and the simulated scattering signals. In the analysis, we extract the Bragg peak position from Gaussian fits of the Bragg peak for every scan. The average value at each delay point is compared to the extracted shift of the simulated Bragg peaks, which match the measured average spectra nicely as seen in Fig. 3(a).

The rise time of the relative Bragg peak shift of about 9 ps corresponds to the time it takes the strain to travel across the photo-induced stress profile determined by the pump absorption profile^59^ [see the inset of Fig. 4(a) and supplementary material].^63^ The analysis further shows that the amplitude of the relative Bragg peak shift is superimposed by modulations of the transient signal, which can be attributed to the propagating strain wave generated by ultrafast laser heating. However, comparing the transient relative peak shift to the strain $\eta =\Delta c/c_{0}$ with lattice constant *c* and $c_{0}=c(t<0\;\text{ps})$ averaged across the entire SL depth [blue line in Fig. 4(b)] reveals substantial differences. The modeled strain response indicates the bipolar strain wave arriving at the SL–substrate interface after 26 ps, where it is partially reflected, resulting in a short modulation of the average lattice strain. In contrast, the experimental peak shift reflects the relative change of the DL thickness across the probing depth of the soft-x-ray light, which is shorter than the entire SL thickness of 157 nm. Consequently, the peak-shift signal probes only the arrival of the reflected strain wave at the surface after 52 ps, while the substrate interface at a depth of 157 nm is not accessible. Moreover, the observed peak shift exceeds the fitted average strain of the Mo/Si SL nearly by a factor of two. Both effects can be understood by comparing the SL peak shift to the weighted averaged strain assuming an exponentially decaying absorption profile of the soft-x-rays [red line in Fig. 4(b)]. We extract this probing depth to be 65 nm, and taking that information depth into account, the resulting weighted strain matches the relative peak shift observed in the experiment as seen in Fig. 4(a).

## III. CONCLUSION

To the best of our knowledge, we presented the first time-resolved diffraction experiment within the water window around 500 eV photon energy at a laser-driven HHG source. The combination of a broadband soft-x-ray source with an energy-dispersive diffractometer enables fast and efficient access to large slices in reciprocal space. We have demonstrated the feasibility of this experimental scheme by probing the first order Bragg peak of a Mo/Si SL both statically and temporally resolved after laser excitation with 40 fs temporal resolution enabled by reasonable short acquisition times facilitating pump-probe experiments. We obtain excellent agreement between the static and transient experimental data with x-ray scattering simulations. In both experiment and theory, the underlying structural information is accessible by analyzing the corresponding Bragg peak shape in reciprocal space. This combined experimental and theoretical approach enables an unambiguous and quantitative determination of the underlying spatiotemporal strain dynamics in the Mo/Si SL after photoexcitation on the femto- and picosecond timescale.

Based on the high stability of the spectral shape of our HHG source, we can follow Bragg peak shifts with remarkable sensitivity. The future implementation of intensity-normalization schemes^60,61^ will enable a new class of experiments that are also sensitive to ultrafast changes of the structure factors associated with nanoscale periodicities. Beyond this experimental proof-of-concept focused on probing the coherent acoustic lattice dynamics in a Mo/Si SL, transient and energy-dispersive diffraction experiments at HHG sources as demonstrated here will be able to target other order phenomena of spin, charge, and orbital origin. This does require reaching the relevant core-to-valence transitions of the elements involved, in order to provide sufficient diffraction contrast. Our state-of-the-art setup reaches the L-edges of the early 3d transition metals, such as Ti and V, as well as the K-edges of O, which already now enables a variety of studies within the broad class of oxide materials. With new powerful laser systems at even longer driver wavelengths at the horizon and further developments in x-ray optics, the L-edges of other, e.g., magnetically relevant elements, such as Mn, Co, Fe, and Ni, will also become within reach for energy-dispersive diffraction experiments at laser-driven HHG sources.^41,62^

## Data Availability

The data that support the findings of this study are available from the corresponding author upon reasonable request.
